# Two years after lockdown: reviewing the effects of COVID‐19 on health services and support for adolescents living with HIV in South Africa

**DOI:** 10.1002/jia2.25904

**Published:** 2022-04-26

**Authors:** Quintin van Staden, Christina A. Laurenzi, Elona Toska

**Affiliations:** ^1^ Department of Sociology University of Cape Town Cape Town South Africa; ^2^ Universitas Hospital Bloemfontein South Africa; ^3^ Institute for Life Course Health Research Department of Global Health Faculty of Medicine and Health Sciences Stellenbosch University Tygerberg South Africa; ^4^ Centre for Social Science Research University of Cape Town Cape Town South Africa; ^5^ Department of Social Policy and Intervention University of Oxford Oxford UK

**Keywords:** adolescents living with HIV, COVID‐19, ART adherence, resource reallocation, barriers, limited support

## Abstract

**Introduction:**

South Africa's progress towards the 95‐95‐95 goals has been significantly slower among adolescents living with HIV (ALHIV), among whom antiretroviral therapy (ART) adherence, retention in care and viral suppression remain a concern. After 2 years of living with COVID‐19, it is important to examine the direct and indirect effects of the pandemic on healthcare resources, access to HIV services and availability of support structures, to assess their impact on HIV care for ALHIV.

**Discussion:**

The COVID‐19 response in South Africa has shifted healthcare resources towards combatting COVID‐19, affecting the quality and availability of HIV services—especially for vulnerable populations, such as ALHIV. The healthcare system's response to COVID‐19 has threatened to diminish fragile gains in engaging ALHIV with HIV services, especially as this group relies on overburdened public health facilities for their HIV care. Reallocation of limited health resources utilized by ALHIV disrupted healthcare workers’ capacity to form and maintain therapeutic relationships with ALHIV and monitor ALHIV for ART‐related side effects, treatment difficulties and mental health conditions, affecting their ability to retain ALHIV in HIV care. Prevailing declines in HIV surveillance meant missed opportunities to identify and manage opportunistic infections and HIV disease progression in adolescents. “Lockdown” restrictions have limited access to healthcare facilities and healthcare workers for ALHIV by reducing clinic appointments and limiting individual movement. ALHIV have had restricted access to social, psychological and educational support structures, including national feeding schemes. This limited access, coupled with reduced opportunities for routine maternal and sexual and reproductive health services, may place adolescent girls at greater risk of transactional sex, child marriages, unintended pregnancy and mother‐to‐child HIV transmission.

**Conclusions:**

Adolescent HIV care in South Africa is often overlooked; however, ART adherence among ALHIV in South Africa is particularly susceptible to the consequences of a world transformed by COVID‐19. The current structures in place to support HIV testing, ART initiation and adherence have been reshaped by disruptions to health structures, new barriers to access health services and the limited available education and psychosocial support systems. Reflecting on these limitations can drive considerations for minimizing these barriers and retaining ALHIV in HIV care.

## 1 INTRODUCTION

Disrupted access to routine HIV centred healthcare is one of the key challenges emerging during the COVID‐19 pandemic globally [1], affecting millions of people living with HIV (PLHIV), especially those within resource‐constrained communities and health systems. Adolescents living with HIV (ALHIV) may experience the adverse effects of the pandemic more acutely, given the complex socio‐emotional, physical and cognitive experiences they face during the second decade of life [2, 3].

Adolescents in South Africa are disproportionately affected by HIV [4, 5, 6, 7], with lower achievement of the 95‐95‐95 HIV cascade [8]. South Africa is home to 20% of all ALHIV globally, with young women disproportionately affected [9]. Despite efforts to prioritize adherence among adolescents in South Africa [10, 11], adolescents experience lower rates of adherence to antiretroviral therapy (ART) compared to other age groups [12, 13, 14, 15]. Even before COVID‐19, ALHIV in South Africa experienced poor coverage of health services and underutilized these available services [16, 17, 18].

The far‐reaching effects of the COVID‐19 pandemic have complicated the health service landscape in South Africa further. Soon after South Africa's first COVID‐19 case in March 2020, the country entered a “state of disaster” and South Africans were instructed to stay home [19, 20, 21]. Varying degrees of lockdown restrictions to prevent the spread of COVID‐19 have limited individual movement and available transport to clinics, and reduced hospitals’ out‐patient services [22, 23, 38, 39].

The consequences of these responses, as well as the financial implications, have affected ALHIV's ability to access HIV services. Health services restructuring linked to COVID‐19 has disrupted services for vulnerable groups [24, 25, 26] and exacerbated pre‐existing barriers to HIV care and support services [6, 17, 18], including for ALHIV. Adolescents have also had to navigate the social and psychological consequences of the pandemic [27, 28] through accompanying national lockdowns [3]. In November 2021, as South African scientists discovered a new, highly transmissible COVID‐19 variant, C.1.2, new concerns emerged about service interruptions in a recovering health system coupled with a slow vaccine rollout [29, 30].

While adolescents have largely avoided direct adverse effects from SARS‐CoV‐2 infection, there are still inadequate data about how living with HIV might increase morbidity and mortality from COVID‐19. A broader understanding of underlying social and structural inequities is also essential for understanding the potential for layered risks and co‐infection among this group. Two years into the pandemic, it is important to reflect on the effects of COVID‐19 on the South African healthcare services delivery and access, and on social and educational support structures, to assess their impact on ART adherence and retention in HIV care among ALHIV.

The public health system, where the majority of ALHIV access HIV services, came under further pressure in the third wave, after the number of cases more than doubled when comparing wave 1 and 2 peaks. After these surges and the subsequent attempts to restore routine HIV services, the public health system has experienced the majority of the COVID‐19 patient load through the fourth wave [31, 33].

While the association of COVID‐19 and adolescents is largely asymptomatic, living with HIV could increase morbidity and mortality and needs to be further assessed. South Africans younger than 20 years old represent the majority of the unvaccinated cohort. In this age‐group, there have been more COVID‐19 infections in the fourth wave, in comparison to the first and second. The percentage of these cases admitted to hospital is the highest since the start of the pandemic [32]. Furthermore, adolescents (10–19) accounted for 9.2% of all COVID‐19 cases since the start of the pandemic. Adolescents with underlying conditions, including HIV, account for 22% of hospital admissions and 60% of deaths [33, 34]. Emerging data from the National Institute for Communicable Diseases show that HIV accounted for 42% of in‐hospital COVID‐19 deaths among South Africans younger than 19 years. Two years into the pandemic, it is important to reflect on the effects of COVID‐19 on the South African healthcare services, and on social and educational support structures, to assess their impact on ART adherence and retention in HIV care among ALHIV.

## 2 DISCUSSION

The response to the COVID‐19 pandemic in South Africa has had direct and indirect effects on structures that promote retention in HIV care for ALHIV. Specifically, it has (1) shifted healthcare resources towards combatting COVID‐19, (2) exacerbated or introduced additional barriers to healthcare and (3) limited access to available support structures for ALHIV. A growing body of research examines the effects of COVID‐19 on HIV services in South Africa more broadly; however, it is important to review specific impacts on ALHIV as a group with unique needs.

### 2.1 Healthcare resource reallocation during COVID‐19

Health system shifts in response to the COVID‐19 pandemic have had significant, direct consequences of the provision of HIV care in South Africa. Biomarker data from the South African National HIV program show a 10‐fold increase in ALHIV aged 15–19 years receiving ART between 2005–2008 and 2015–2016, with an estimated 66% of all diagnosed ALHIV receiving ART by 2016 [8]. Recent modelling, however, shows that these gains will be halted by COVID‐19‐affected healthcare changes and ensuing lockdowns [35].

This progress has been jeopardized, in part, by reallocating resources away from HIV care [36]. Healthcare workers in the public sector were shifted from routine HIV service provision to help manage the pandemic—including 28,000 HIV community workers redeployed to assist with COVID‐19 screening and testing [37]. While health workers have had to spend less time providing HIV counselling and ART adherence support [13], they have also faced the new demands of managing COVID‐19, adding strain to an already‐overburdened workforce. This potential burnout may affect health workers’ ability to provide empathic care and form therapeutic relationships, which are critical to retaining ALHIV in HIV care [38, 39]. As pandemics persist, burdens on vulnerable populations are exacerbated disproportionately in countries like South Africa with overburdened public healthcare systems serving poorer populations, contrasting with well‐resourced private healthcare for a minority [40, 41]. These countries’ health systems may be less‐equipped to manage multiple competing health crises, such as HIV and COVID‐19. Global data during the COVID‐19 pandemic, including from China and Uganda, have shown that HIV patients are at risk of ART discontinuation [42, 43].

Importantly, these trends may be more pronounced for adolescent patients. The majority of ALHIV use South Africa's public health services, which have also borne significant care provision for COVID‐19 patients during the country's four COVID‐19 waves [32, 44, 45, 46]. While early studies in 2020 showed promising trends in retaining patients on ART [47], national data show a dramatic decline in important HIV viral load and CD4 surveillance testing [48, 49]. Results were inconsistent among ALHIV: ART collections and HIV clinic visits among ALHIV were largely unaffected by South Africa's initial lockdown [47, 50]. The National Health Laboratory confirmed this decline, detailing a 33% decrease in testing for CD4 cell count (a test performed on the day of HIV diagnosis), between March and April 2020 (spanning the first lockdown) [51]. Combined with a reported 22% decline in viral load testing and a significant reduction in testing for cryptococcal antigen (a marker of advanced HIV disease in South Africa [48]), reductions in TB testing [49] indicate changes in HIV care provision and potentially HIV‐related outcomes, even if not in routine and ART collection visits.

Interestingly, the same study documented a 60% decline in HIV testing and ART initiation in the 15–24 years age‐group, compared to 47.6% across all age groups [47, 50]. Among sub‐Saharan African countries, South Africa had among the most significant declines in HIV testing in children and adolescents in the first months of the initial, strictest lockdown. These declines meant that adolescents living with undiagnosed HIV were unable to link to HIV care and initiate treatment [52]. Planned task‐shifting from HIV services, and difficulties engaging with HIV care due to lockdown restrictions—particularly in urban areas—may have contributed to this decline [47, 53] Together, these data suggest that while diagnosed and ALHIV may have continued receiving some elements of HIV care, the health system's ability to detect and respond to new infections and support ALHIV at risk of developing AIDS was substantially diminished—risking fragile gains in ALHIV care.

Importantly, shifts in health service provision also resulted in downstream effects on other health outcomes, including heightened risks of unintended pregnancies for adolescent girls [54, 55]. Infants born to adolescent girls under age 18 accounted for 33.8% of all births registered in South Africa in 2020 [56]. Qualitative data have revealed inadequate sexual and reproductive health service provision during the pandemic, disrupting continued contraceptive access for many adolescent girls [57]. Recent data have also shown a decline in HIV antenatal testing among South African women, and fewer pregnant mothers living with HIV receiving ART during the pandemic [58, 59]. Adolescent girls are much likelier than older mothers to have unintended pregnancies, and have three times the rate of mother‐to‐child HIV transmission [60]; many also first learn of a positive status through their antenatal visits [61]. Fewer routine visits may translate to missed opportunities for identifying new infections and pregnancies, and delay initiation into antenatal care. Additional modelling suggests that disruptions such as COVID‐19 in HIV services can lead to a two‐fold increase in mother‐to‐child transmission of HIV [62] and lead to significant maternal deaths [63].

### 2.2 Additional barriers to accessing HIV care services

The continuation of HIV healthcare services and adherence support for ALHIV is critically important, and understanding access barriers from the patient side is critical to gauging the broader impact of COVID‐19 [64]. The pandemic has created additional barriers for ALHIV seeking to access HIV services, raising fears of COVID‐infection [65], heightening stigma associated with COVID‐19 [66] and prioritizing patients with COVID‐19 symptoms [29]. A large survey of South Africans found that 57% of participants were apprehensive to attend a health facility during lockdown [37]. Furthermore, young ALHIV reliant on caregivers to accompany them to clinic visits may be less likely to receive this support, as older or at‐risk caregivers avoided health facilities [68].

Responding to these challenges, strategies such as multi‐month dispensing and differentiated service delivery programs to curb ART interruption have provided flexible responses to ensuring that patients collect their ART [69]. However, ART needs to be taken regularly and correctly; multi‐month dispensing, while requiring fewer routine clinic visits [70], may not work as well for ALHIV, who often require more active support and monitoring to ensure adherence and retention in care. These active interventions support ALHIV who experience ART‐related side effects, or difficulty with high pill burdens and swallowing their medication, which in turn makes them likelier to stop taking their ART [12, 71]. Missed clinic appointments are independently associated with an increased risk of acquiring an AIDS‐defining illness [72].

Furthermore, COVID‐19 has affected both the quantity and quality of HIV care that health facilities can provide, leading to the breakdown in HIV testing and continuation of care. Reduced contact frequency, more personal protective equipment and limited client‐provider visit duration [67] have prevented adolescents from engaging in important services, reducing access to critical knowledge and counselling needed for their HIV care and limited time to build trust with providers [46]. For example, the “client” and “provider‐initiated” HIV testing algorithm, requiring repeated pre‐ and post‐test HIV counselling, has been temporarily sidelined by the social restrictions enforced during lockdown and new hospital COVID‐19 protocols [73]. Interruptions in adherence counselling may also affect ALHIV more significantly, as ALHIV are likelier to enter new relationships and struggle to practice safe sex, leading to a cascade effect of increased viral loads, HIV transmission and re‐infection. After 2 years of COVID‐19, these transmission prevention protocols are still consistently adhered to by healthcare providers––maintaining learnt behavioural tailoring, despite restrictions loosening [74, 75].

The pandemic has also placed social and economic strain on South African households [76]. A nearly 40% increase in unemployment among South African adults between February and April 2020 led to approximately 3 million South Africans falling below the poverty line [76], with a slow rate of recovery encumbered by multiple COVID‐19 waves. Chronic financial stress may impair caregivers’ ability to provide support to adolescents, limiting transport funds for clinic visits, and leading to poorer clinical attendance and risk of poor viral suppression [10, 77, 78].

### 2.3 Limited support structures to support HIV outcomes for ALHIV

In addition to health system shifts and additional barriers to accessing HIV services, ALHIV have experienced limited access to social and educational support structures [24, 79, 80, 81]. Among ALHIV, strong social support networks, such as school attendance, community involvement, peer support and nutritional support, facilitate ART adherence and retention in care [82]. These evidence‐based support structures were less accessible to adolescents during the most restricted lockdown periods [83, 84, 85].

The COVID‐19 pandemic has had a disproportionate effect on the poorest individuals and communities, with significant psychosocial and financial impacts that lead to poorer ART adherence [86]. Almost half of respondents in a nationally representative survey reported running out of money for food in the first month of lockdown, with people living in informal settlements suffering the greatest financial distress [87]. Adolescents also reported food insecurity and hunger during the pandemic, both of which can affect ALHIV's ability to adhere to ART [27, 88]. There are growing concerns that these conditions may have facilitated transactional sex and early child marriages, with adolescent girls more affected than boys [54, 89, 90].

South African schools closed in March 2020 during the first national lockdown, and only reopened in June with varying protocols of limited numbers and rotational schedules [79]. These closures and access restrictions left many adolescents without access to school‐provided psychological, social and nutritional support [24], including over 9 million South African students supported by the National School Nutrition Programme [91].

Mental health in ALHIV is often overlooked and undervalued to support adherence. A significant portion of ALHIV experience internalized stigma and continue to feel discriminated against [92, 93]. Moreover, mental health conditions are common in ALHIV [94]. Psychosocial interventions targeting ALHIV that may improve ART adherence and lead to a decrease in HIV viral load [95] may not be easily accessible during this pandemic. Since adolescents may face an increased risk of mental health problems during the pandemic [24], there is a greater possibility of conditions, such as depression, which could further complicate future adherence in ALHIV [96].

## 3 CONCLUSIONS

The direct and indirect consequences of COVID‐19 threaten South Africa's progress in achieving the “95‐95‐95” goals relating to the HIV care cascade. HIV care among ALHIV is influenced by complex interactions between individuals, their environment and the health system. Adolescents are particularly vulnerable to these consequences, and their ability to access comprehensive HIV care, including ART, is essential to their ability to live long and healthy lives.

The persistence of significant COVID‐19 waves 2 years later, along with vaccine hesitancy, underlines the importance of reviewing HIV testing, ART initiation and adherence, and ultimately retention in care among ALHIV as COVID‐19 will continue to be a part of the lives of South Africa's ALHIV for the foreseeable, unpredictable future. ALHIV need to be taken care of and their ART adherence and retention in HIV care be made a priority, especially in the context of less frequent engagement with health services due to multi‐month ART dispensing approaches.

## COMPETING INTERESTS

There are no competing interests to declare.

## AUTHORS' CONTRIBUTIONS

QVS: Primary author, substantial contribution to drafting the paper, critical revision and approval to submit. CL and ET: Substantial contribution to drafting the paper, critical revision and approval to submit.

## FUNDING

None of the authors were funded to produce this commentary.

## Data Availability

The data used to support this commentary are included within the article.
